# Increasing uptake of colorectal cancer screening in Korea: a population-based study

**DOI:** 10.1186/1471-2458-10-265

**Published:** 2010-05-21

**Authors:** Kui Son Choi, Jae Kwan Jun, Hoo-Yeon Lee, Myung-Il Hahm, Jae Hwan Oh, Eun-Cheol Park

**Affiliations:** 1 National Cancer Control Institute, National Cancer Center, 111, Jungbalsan-ro, Ilsandong-gu, Goyang-si, Gyeonggi-do, Korea; 2 Department of U-Healthcare Management, College of Medical Science, Soonchunhyang University, Chungnam, Korea; 3 Research Institute and Hospital, National Cancer Center, 111, Jungbalsan-ro, Ilsandong-gu, Goyang-si, Gyeonggi-do, Korea

## Abstract

**Background:**

Colorectal cancer (CRC) screening rates are low in most Asian countries and remain largely unknown. This study examined trends in CRC screening rates after the introduction of the Korean National Cancer Screening Programme (NCSP) and determined the factors associated with uptake of CRC screening by test modality over time.

**Methods:**

An annual population-based survey conducted through nationally representative random sampling from 2005-2008. In total, 3,699 participants from the 2005-2008 surveys were selected as study subjects. Face-to-face interviews were performed to assess the utilization rate of CRC screening by each screening modality.

**Results:**

Overall, CRC screening within the recommended time interval increased significantly from 22.9% in 2005 to 36.6% in 2008 (*p *< 0.001). The proportion of subjects receiving a fecal occult blood test (FOBT) test within the previous year increased significantly from 7.2% in 2005 to 21.3% in 2008 (*p *< 0.001). Increases in FOBT testing were highest among those who had a lower income status (relative difference = 511.9%) and women (relative difference = 266.1%). Endoscopy use also increased from 18.0% in 2005 to 20.5% in 2008, albeit not significant. Overall, those who were male, non-smokers, 60-69 years old, and had a higher income status were more likely to have undergone up-to-date endoscopy and CRC screening.

**Conclusions:**

This study revealed a substantial increase in up-to-date CRC screening in the general population from 2005 to 2008. However, more than half of adults in Korea are still not up-to-date with their CRC tests. It will be important to continue to investigate factors associated with up-to-date CRC screening by each modality.

## Background

Worldwide, colorectal cancer (CRC) is the fourth most commonly diagnosed cancer in men and third in women [1]. About 1 million new cases of CRC were diagnosed in 2002 (9.4% of all cancer diagnoses worldwide) [1]. However, at least a 25-fold variation in the occurrence of CRC has been reported worldwide. Western countries have the highest incidence rates [2]. Recently, CRC incidence rates have been increasing rapidly in countries where the overall risk was once low (especially in Asia), whereas rates in high-risk countries are either increasing gradually, stabilizing (North and West Europe), or declining over time (North America) [3]. In Korea, incidence rates have risen markedly over the past few years (e.g., annual percent change, APC = 7.3% per year between 1999 and 2005) [4,5]. From 2003 to 2005, CRC was the third most commonly diagnosed cancer in Korea (12.0% of all cancer diagnoses) [5].

Based on evidence that screening reduces CRC incidence and mortality [6-9], national guidelines in several countries now recommend regular CRC screening for average-risk persons aged ≥50 years using one or more of the following options: annual fecal occult blood test (FOBT), flexible sigmoidscopy every 5 years, a combination of FOBT (or fecal immunochemical test) and flexible sigmoidoscopy (FSIG), colonoscopy every 10 years, and/or double-contrast barium enema (DCBE) every 5 years [10,11]. Although the incidence of CRC is increasing rapidly in Asian countries, national guidelines for CRC screening are nonexistent in most Asian countries [12]. Recently, the Asia Pacific Working Group on Colorectal Cancer reached a consensus to develop guidelines for CRC screening and recommend FOBT, FISG, and colonoscopy as the best options [13].

In 2002, the National Cancer Center in Korea, and Korean Society of Coloproctology developed guideline for CRC screening in average-risk adults [14]. This guideline recommends that average-risk adults begin screening for CRC at age 50, having either (a) a colonoscopy every 5-10 years or (b) DCBE combined with FSIG every 5-10 years. In 2004, the Korean government also introduced nationwide CRC screening as part of the National Cancer Screening Programme (NCSP) for low-income groups. Due to capacity related to performing colonoscopy screenings on every age-eligible person, the NCSP provides an annual FOBT (immunochemical) test as the initial mass screening method for men and women ≥50 years instead of colonoscopy [15]. The NCSP provides further investigation with a colonoscopy or DCBE for any low-income individual with a positive FOBT result. In 2004, Medical Aid recipients and National Health Insurance (NHI) beneficiaries within the lower 30% income bracket were eligible for free-of-charge FOBT screening services for CRC under NCSP. In 2005, the NCSP expanded its target population to the 50% income bracket. Apart from these organized CRC screening programmes, FOBT, DCBE, and colonoscopy testing are conducted in outpatient clinics or private health-assessment centers for opportunistic screening. However, in these cases individuals must pay all procedure-related costs.

Despite the evidence that screening can reduce the incidence of and mortality from CRC, uptake of CRC screening has been lower than for other mass cancer screening interventions [16,17]. Globally, only half the eligible population undergoes CRC screening [18-22]. In particular, screening for CRC is not commonly practiced in most Asian countries, and CRC screening behaviors in Asian populations remains largely unknown. Although national CRC screening programms have been offered since 2004 in Korea, no studies have reported the extent to which various CRC screening procedures have been used by the general population. In addition, no studies have analyzed whether usage of various types of screening procedures has changed over time since the introduction of the national CRC screening programme.

This study assessed the rates of CRC screening in Korea by screening modalities using a population-based survey. In particular, it examined trends in CRC screening rates after the introduction of the NCSP using four independent cross-sectional, nationally representative datasets collected during 2005-2008, and identified the factors associated with uptake of CRC screening by test modality.

## Methods

### Study Population

This study was based on the 2005, 2006, 2007, and 2008 Korean National Cancer Screening Survey (KNCSS). The KNCSS is an annual cross-sectional survey that uses a nationally representative random sampling to investigate Korean participation rates in cancer screening for five common cancers: gastric, liver, colorectal, breast, and cervical [23]. In each study year, women aged ≥30 years old and men aged ≥40 years old were selected from the Resident of Registration Population data of the Korea National Statistical Office using a stratified, multistage, and random sampling procedure according to geographic area, age, and gender. Face-to-face interviews were performed.

**Figure 1:**
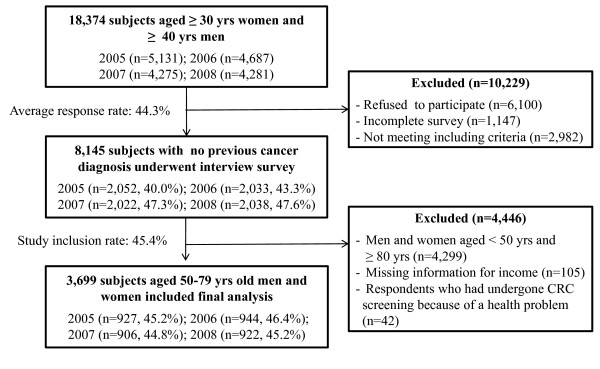
**Participant selection process**.

In 2005, a total of 2,052 men (aged ≥40 years) and women (aged ≥30 years) completed the interview (response rate, 40.0%); in 2006, 2,033 (response rate, 43.4%), in 2007, 2,022 (response rate, 47.3%), and in 2008, 2,038 subjects (response rate, 47.6%) completed the interview (Figure 1). Of the respondents, this study analyzed data from cancer-free male and female subjects aged ≥50 years, the NCSP's recommended starting age for CRC screening among persons at average risk [15]. Further, respondents (n = 42) who had undergone CRC screening because of a health problem or as a follow-up to a previously identified colorectal problem were excluded from the analysis since the focus of the study is on routine screening. In total, 3,699 participants from the 2005-2008 surveys were selected as study subjects. This study was approved by the institutional review board of the National Cancer Center, Korea.

### Measures

Questions were developed to assess the utilization rate of CRC screening by each screening modality. Questions were prefaced with a two-to-three-sentence description of the tests to help respondents differentiate among the tests. The following main distinguishing characteristics of the tests were provided: (a) FOBT is a test to find blood using your stool sample. This test may use a special kit at home or container to store your stool and send it to a medical center; (b) endoscopy (e.g., sigmoidoscopy and colonoscopy) is performed with a flexible tube passed through the anus (we did not distinguish between colonoscopy and sigmoidoscopy because lay people usually do not differentiate between these two tests.); (c) DCBE is a radiological exam that looks for polyps or cancer in the colon or rectum. During this procedure, a physician administers a liquid with barium through the anus and into the rectum and colon and takes x-rays.

Previous CRC screening experience was assessed by asking respondents: (a) whether they had ever been screened for CRC, (b) when they underwent their most recent screening, and (c) which tests they underwent during their most recent CRC screening (FOBT, endoscopy, or DCBE). A respondent was considered to be "up-to-date" if he or she had undergone an FOBT test within the past 1 year, an endoscopy test within the past 10 years, or a DCBE test within the past 5 years.

This survey also included demographic and socioeconomic factors (e.g., gender, age, education, and household income) associated with the uptake of CRC screening. Household income was categorized into three groups based on monthly post-tax household income (US$1 = 1000 won). We also included questions about health insurance status. In Korea, every citizen, with the exception of some medical aid beneficiaries, is insured by the NHI programme. Thus, subjects were classified as "NHI beneficiary" or as "medical aid beneficiary." With regard to health behavior, we ascertained smoking status. Subjects were classified as current smokers if they reported current smoking for at least 1 year or as non-smokers if they had never smoked or had previously smoked but had not smoked for at least the past 1 year. Health status was measured by self-report; respondents rated their general health status on a five-point scale (1 = very poor, 5 = excellent).

### Statistical analysis

Descriptive statistics were computed for all socioeconomic factors and health behaviors. We calculated CRC screening rates from 2005 to 2008 for each screening method. First, we weighted samples by the appropriate weight based on age and gender from the 2005 Korean standard population [24]. Age- and gender-adjusted logistic regression models were used to assess the odds of having CRC screening by FOBT, endoscopy, and any test within the guidelines. Further trends were examined using the Cochran-Armitage trend test.

Changes in CRC testing between 2005 and 2008 were assessed by comparing the rates from 2005 with the rates in 2008 as relative risks. These risks are reported as the percentage changes ([relative risk - 1] × 100) in rates between the two years. Percentages were compared using Fisher's exact test.

Multivariate logistic regression was used to determine the factors associated with CRC screening procedure use by FOBT, endoscopy, any test within guidelines. Initially, separate models were tested for each outcome from the 2005-2008 survey samples, followed by models that included all the samples with time as an indicator variable. Because the results were similar when using separate models versus one, we focused the analyses on combined models. We did not include health insurance type as a variable in the final multivariate model because of its strong correlation with monthly household income. To maintain a family α of 0.05 for the analyses of the 3 outcomes, we employed a Bonferroni adjustment using α = 0.05/3 = 0.017. All statistical analyses were conducted using SAS statistical software (version 9.1; SAS Institute Inc., Cary, NC).

## Results

Table 1 presents descriptive data about the study participants. The demographic distribution of the sample was almost homogenous among survey years, with the exception of household income and health insurance status. A larger proportion of 2008 survey respondents reported higher income compared to 2005 survey respondents.

**Table 1:** Characteristics of respondents, 2005-2008 (n = 3,699)

	2005 (n = 927)	2006 (n = 944)	2007 (n = 906)	2008 (n = 922)	*P *value
					
	%	%	%	%	
Gender					
Male	46.8	46.9	47.1	47.3	0.997
Female	53.2	53.1	52.9	52.7	
Age (years)					
50-59	47.8	48.4	48.8	49.3	0.972
60-69	33.6	32.6	31.8	31.3	
70-79	18.6	19.0	19.4	19.4	
Education (level)					
Did not complete high school	62.9	63.1	60.3	59.1	0.153
High school graduate or above	37.1	36.4	39.7	40.9	
Monthly household income (US$)					
Less than 1,500	24.7	27.1	21.4	20.9	0.002
1,500-3,000	55.1	48.6	52.1	51.2	
3,000 or more	20.2	24.3	26.5	27.9	
Health insurance type					
Medical aid	5.1	7.8	4.1	5.3	0.005
National Health Insurance	94.9	92.2	95.9	94.7	
Self-reported health status					
Fair/poor	16.8	15.8	15.2	12.7	0.277
Good	28.7	28.0	27.8	28.3	
Excellent/very good	54.5	56.2	57.0	59.0	
Smoking status					
Never smoked	23.9	23.1	22.7	20.3	0.391
Former smoker	14.8	13.1	14.6	16.1	
Current smoker	61.3	63.8	62.7	63.6	

Table 2 presents the proportion of subjects who reported CRC testing. The proportion reporting FOBT testing within the previous year increased significantly from 7.2% in 2005 to 21.3% in 2008 (*p *< 0.001). Rates of endoscopy use also increased from 18.0% in 2005 to 20.5% in 2008, albeit not significant (*p *= 0.060). Overall, CRC screening within the recommended time interval increased significantly from 22.9% in 2005 to 36.6% in 2008 (*p *< 0.001).

**Table 2:** Colorectal cancer screening rates in 2004, 2005, 2006, and 2008.

	FOBT within previous year		Endoscopy^a ^within previous 10 years		Any test^b ^within recommended time interval	
			
	Percent	OR (95% CI)^c^	Percent	OR (95% CI)^c^	Percent	OR (95% CI)^c^
2005	7.2	1.00 (referent)	18.0	1.00 (referent)	22.9	1.00 (referent)
2006	13.1	2.15 (1.57-2.96)	16.5	0.94 (0.74-1.19)	26.8	1.32 (1.07-1.64)
2007	17.5	3.03 (2.23-4.12)	18.0	1.06 (0.83-1.34)	29.0	1.46 (1.18-1.80)
2008	21.3	3.68 (2.72-4.97)	20.5	1.21 (0.96-1.53)	36.6	2.02 (1.64-2.48)

*P*_*trend*_^d^		<0.001		0.060		<0.001

^a ^Flexible sigmoidoscopy or colonoscopy.

^b ^Includes FOBT test within the past one year, endoscopy test within the past 10 years, or DCBE test within the past 5 years. The number screened does not equal the sum of participants because people may have had more than one test.

^c ^Age and gender adjusted logistic regression models for each outcome were estimated.

^d ^*p*-value for trend calculated using continuous values.

Tables 3 show changes in FOBT and endoscopy uses between 2005 and 2008, comparing 2005 rates with 2008 rates as relative risks. The results indicate an increased proportion of FOBT use from 2005 to 2008 in all study population. In particular, increases in FOBT testing were highest among those with lower income status (relative difference percentage = 511.9%) and women (relative difference percentage = 266.1%).

**Table 3:** Rate of up-to-date fecal occult blood test and endoscopy

	FOBT within the previous year					Endoscopy^a ^within the previous 10 years				
		
			2008 vs 2005					2008 vs 2005		
						
	2005 %	2008 %	Relative Difference %^b^	Absolute Difference *percentage points*	P value^c^	2005 %	2008 %	Relative Difference %^b^	Absolute Difference *percentage points*	P value^c^
Gender										
Male	8.7	21.0	141.4	12.3	<0.001	18.2	19.3	6.0	1.1	0.756
Female	5.9	21.6	266.1	15.7	<0.001	17.8	21.6	21.3	3.8	0.156
Age (years)										
50-59	6.4	19.4	203.1	13.0	<0.001	17.1	21.3	24.6	4.2	0.132
60-69	7.7	22.5	192.2	14.8	<0.001	19.9	21.5	8.0	1.6	0.675
70-79	8.4	24.0	185.7	15.6	<0.001	16.9	16.6	-1.8	-0.3	0.938
Education (level)										
Did not complete high school	6.9	22.4	224.6	15.5	<0.001	17.7	19.6	10.7	1.9	0.449
High school graduate or above	7.7	19.7	155.8	12.0	<0.001	18.4	21.7	17.9	3.3	0.308
Monthly household income (US$)										
Less than 1,500	4.2	25.7	511.9	21.5	<0.001	15.8	17.0	7.6	1.2	0.841
1,500-3,000	8.5	19.2	125.9	10.7	<0.001	17.1	18.9	10.5	1.8	0.516
3,000 or more	7.5	21.8	190.7	14.3	<0.001	22.9	26.0	13.5	3.1	0.521
Self-reported health status										
Fair/poor	5.1	13.1	156.9	8.0	0.0184	17.1	17.9	4.7	0.8	0.987
Good	6.0	21.5	258.3	15.5	<0.001	18.2	24.4	34.1	6.2	0.104
Excellent/very good	8.5	23.0	170.6	14.5	<0.001	18.1	19.1	5.5	1.0	0.719
Smoking status										
Current smoker	6.6	20.7	213.6	14.1	<0.001	15.2	19.3	27.0	4.1	0.346
Former smoker	6.7	22.1	229.9	15.4	<0.001	22.8	19.3	-15.4	-3.5	0.575
Never smoked	7.5	21.3	184.0	13.8	<0.001	17.9	21.1	17.9	3.2	0.183

^a ^Flexible sigmoidoscopy or colonoscopy.

^b ^The percentage changes ([relative risk - 1] × 100) in the rates between 2005 and 2008.

^c ^All *P *values are two-sided and were calculated for exact comparisons of the rates between 2005 and 2008.

The results from the full logistic regression models for FOBT (model 1), endoscopy (model 2), and any test (model 3) are shown in Table 4. Rates of FOBT testing and any CRC testing were significantly increased in 2008 compared to 2005 at the adjusted α of 0.017. With regard FOBT test, those who aged 60-69 years, and reported their health status as "excellent/very good" were more likely to have undergone FOBT testing. Endoscopies were significantly more likely among those aged 60-69 years, those with higher income, and non-smokers. Women were significantly less likely to have had an endoscopy test. Overall, male, individuals aged 60-69 years, those with higher income, and non-smokers were more likely to have undergone up-to-date CRC screening.

**Table 4:** Full Regression Models for Receipt of FOBT, Endoscopy, and Any test

	Model 1: FOBT			Model 2: Endsoscopy^a^			Model 3: Any test^b^		
			
	aOR	95% CI	*P*	aOR	95% CI	*P*	aOR	95% CI	*P*
Gender									
Male	1.00	Referent		1.00	Referent		1.00	Referent	
Female	0.84	0.64 - 1.11	0.212	0.73	0.57 - 0.93	0.011	0.72	0.58 - 0.90	0.003
Age, y									
50 - 59	1.00	Referent		1.00	Referent		1.00	Referent	
60 - 69	1.38	1.12 - 1.70	0.003	1.45	1.20 - 1.76	0.001	1.49	1.26 - 1.75	<0.001
70 - 79	0.94	0.67 - 1.34	0.787	0.91	0.65 - 1.27	0.591	0.90	0.69 - 1.22	0.471
Education (years)									
Did not complete high school	1.00	Referent		1.00	Referent		1.00	Referent	
High school graduate or above	1.00	0.80 - 1.24	0.984	1.09	0.90- 1.33	0.390	1.03	0.87 - 1.22	0.727
Monthly household income (US$)									
Less than 1,500	1.00	Referent		1.00	Referent		1.00	Referent	
1,500 - 3,000	0.97	0.75 - 1.24	0.797	1.27	1.00 - 1.61	0.053	1.13	0.92 - 1.37	0.241
3,000 or more	1.11	0.83 - 1.49	0.489	1.81	1.37 - 2.38	<0.001	1.51	1.19 - 1.91	0.001
Self-reported health status									
Fair/poor	1.00	Referent		1.00	Referent		1.00	Referent	
Good	1.41	1.01 - 1.96	0.044	0.87	0.67 - 1.14	0.319	1.05	0.82 - 1.33	0.702
Excellent/very good	1.53	1.12 - 2.08	0.007	0.79	0.61 - 1.01	0.064	1.02	0.82 - 1.27	0.859
Smoking status									
Current smoker	1.00	Referent		1.00	Referent		1.00	Referent	
Former smoker	1.25	0.92 - 1.69	0.153	1.06	0.79 - 1.42	0.698	1.23	0.96 - 1.57	0.096
Never smoked	1.21	0.89 - 1.64	0.225	1.56	1.19 - 2.06	0.002	1.54	1.21 - 1.95	0.001
Time									
2005	1.00	Referent		1.00	Referent		1.00	Referent	
2006	2.13	1.55 - 2.93	<0.001	0.92	0.72 - 1.17	0.508	1.31	1.06 - 1.62	0.014
2007	2.98	2.19 - 4.06	<0.001	1.02	0.80 - 1.29	0.900	1.42	1.15 - 1.75	0.001
2008	3.57	2.64 - 4.83	<0.001	1.16	0.91 - 1.47	0.204	1.94	1.58 - 2.40	<0.001

-2 log likelihood		3005.931			3502.828			4375.334	

^a ^Flexible sigmoidoscopy and colonscopy.

^b ^Includes FOBT and/or endoscopy. The number screened does not equal the sum of participants (i.e., people may have had > 1 test).

## Discussion

Early detection and treatment is an important way to reduce mortality from colorectal cancer. Nonetheless, Asians tend to have little understanding about CRC screening. This report is the first to assess CRC screening rates by various screening methods in a Korean population at average risk for the disease. Data from national surveys reveal that CRC screening remains underused in Korea: in 2008, only 36.6% of the Korean population aged ≥50 years had undergone CRC screening within the recommended time interval. In contrast, data from the National Health Interview Survey indicate that in the United States, 44% of adults aged 50 to 64 years had undergone a recommended CRC screening test in 2005 [25].

However, the current analysis revealed a substantial increase in up-to-date CRC screening in the general population from 2005 to 2008. In addition, it showed substantial changes in the proportion of the general population being screened by different screening modalities. In 2005, only 23% of the Korean population was screened by FOBT, endoscopy (colonoscopy and/or sigmoidoscopy), and/or DCBE within guidelines. Screening rates did increase over the 3-year period, yet by 2008 still only 36.6% of respondents had been screened by 'any test' within guidelines. The majority of this increase was explained by a 14% increase in screening FOBT, yet there was only a 2.5% increase in endoscopy. Although endoscopy testing increased only slightly between 2005 and 2008 (relative difference percentage = 14%), FOBT testing rates increased relatively more during the same period (relative difference percentage = 196%). By 2008, FOBT had become the most common CRC screening test modality. In contrast, in the United States the prevalence of CRC screening using endoscopy was more than twice that using FOBT [25].

The large increases in FOBT use can be explained in part by health care policy and public awareness. Since 2004, the Korean government and NHIC have provided free-of-charge annual FOBT testing as an initial screening tool for low-income individuals. The current analysis revealed that the greatest increases in FOBT use were among individuals with low household income (relative difference percent = 512%). Further, FOBT testing increased 266% among women from 2005 to 2008. These results suggest that the substantial increase in FOBT testing among women and low-income groups might have been associated with the introduction of the NCSP. The current analysis also revealed that endoscopy screening rates increased slightly from 2005 to 2008. These relatively small increases in endoscopy use can be attributed in part to the fact that the NHI only reimburses colonoscopy and sigmoidscopy testing for those with symptoms or colorectal problems. Other health or educational policies, e.g., mass-media campaigns might have impacted on the increment of the CRC screening rates.

Consistent with previously published literature regarding CRC screening, we have shown that test use varies by gender, age, income, smoking status [16,18-22]. Interestingly, respondents with higher income levels were more likely to have an endoscopy test. Under the Korean health insurance system, an endoscopy test costs almost 10 times as much as an FOBT test. In addition, the NSCP ensures free-of-charge FOBT testing for all individuals, whereas endoscopy testing is only provided free of charge for those whose FOBT results are positive. Furthermore, the NHI only reimburses colonoscopy and sigmoidscopy testing for those with symptoms or colorectal problems. Therefore, endoscopy use varied with household income, possibly suggesting that the cost of endoscopy is a barrier to CRC screening.

We examined trends in screening for CRC by screening modality and explored the factors associated with uptake of CRC screening in a population sample. However, the study's cross-sectional design and reliance on self-reported screening limit the KNCSS. Previous studies have indicated that self-reported FOBT screening data might result in overestimates of the proportion screened [26,27], and recent research has suggested that over-reporting may be greater for FOBT than for endoscopy [28]. Therefore, the proportion of subjects who were actually up-to-date for CRC testing may be lower than that indicated by the current analysis. Further, the cross-sectional design of the study precludes conclusions about whether any observed associations were causal. Finally, our study sample may not be representative because of low response rate. However, the KNCSS respondents had health characteristics and behaviors that were very similar to those found in other extensively used surveys, such as the Korea National Health and Nutrition Survey.

## Conclusion

Our results demonstrate that CRC screening increased after the introduction of the NCSP, according to a population-based survey. Increasing recognition of the efficacy and importance of CRC screening might have influenced CRC screening rates. However, overall screening rates increased less than did FOBT screening rates. The data indicate increases in both FOBT and endoscopy testing between 2005 and 2008, combined with a shift toward greater use of FOBT compared with endoscopy. This shift might be associated with national CRC screening policies. The introduction of free CRC screening appears to have been effective in increasing CRC screening rates among low-income groups in Korea. However, the increases in FOBT test use have implications for public health practice. Although FOBT is simple, safe, most inexpensive, and its routine use was shown to reduce CRC mortality, it is limited by poorer sensitivity, mainly for premalignant lesions. Colonoscopy, on the other hand, is a more accurate technique, yet invasive, carries a risk of bleeding and perforation, requires preparation and premedication, and involves much higher costs. The options for CRC screening tests allow for flexibility but can also render decisions about recommending or choosing a particular test difficult. Each test has its tradeoffs in terms of efficacy, complications, discomfort, time, and cost [20]. More than half of adults in Korea are still not up-to-date with their CRC tests. It will be important to continue to monitor trends in screening, as well as to investigate factors associated with up-to-date CRC screening. More research will also be required to increase CRC screening rates, particularly among those who had not have CRC screening under NCSP.

## Abbreviations

(CRC): Colorectal Cancer; (NCSP): National Cancer Screening Programme; (FOBT): Fecal occult blood test; (FSIG): Flexible sigmoidoscopy; (DCBE): Double-contrast barium enema; (KNCSS): Korean National Cancer Screening Survey.

## Competing interests

The authors declare that they have no competing interests.

## Authors' contributions

KSC contributed to the conception of study and interpretation, and writing the manuscript. JKJ and HYL participated in the conception of the study and performed the statistical analysis. MIH participated in the design and data collection. SL contributed to the drafting of the manuscript. JHO and ECP participated in the design and coordination of the study. All authors read and approved the final manuscript.

## Pre-publication history

The pre-publication history for this paper can be accessed here:

http://www.biomedcentral.com/1471-2458/10/265/prepub
